# mTOR, a new potential target for chronic pain and opioid-induced tolerance and hyperalgesia

**DOI:** 10.1186/s12990-015-0030-5

**Published:** 2015-05-30

**Authors:** Brianna Marie Lutz, Sam Nia, Ming Xiong, Yuan-Xiang Tao, Alex Bekker

**Affiliations:** Rutgers Graduate School of Biomedical Sciences, New Jersey Medical School, Rutgers, The State University of New Jersey, Newark, NJ 07103 USA; Department of Anesthesiology, New Jersey Medical School, Rutgers, The State University of New Jersey, Newark, NJ 07103 USA

**Keywords:** Pain, NMTOR, Opioid, Hyperalgesia, Tolerance

## Abstract

Chronic pain is a major public health problem with limited treatment options. Opioids remain a routine treatment for chronic pain, but extended exposure to opioid therapy can produce opioid tolerance and hyperalgesia. Although the mechanisms underlying chronic pain, opioid-induced tolerance, and opioid-induced hyperalgesia remain to be uncovered, mammalian target of rapamycin (mTOR) is involved in these disorders. The mTOR complex 1 and its triggered protein translation are required for the initiation and maintenance of chronic pain (including cancer pain) and opioid-induced tolerance/hyperalgesia. Given that mTOR inhibitors are FDA-approved drugs and an mTOR inhibitor is approved for the treatment of several cancers, these findings suggest that mTOR inhibitors will likely have multiple clinical benefits, including anticancer, antinociception/anti-cancer pain, and antitolerance/hyperalgesia. This paper compares the role of mTOR complex 1 in chronic pain, opioid-induced tolerance, and opioid-induced hyperalgesia.

## Introduction

Chronic pain and its related sequelae represent a severe public health challenge affecting nearly 30 % of the population [1]. In addition to its impact on quality of life, chronic pain has an economic impact, costing over $560 billion annually [2]. Opioids have been the mainstay of chronic pain treatment for many decades despite a host of shortcomings including tolerance and hyperalgesia, which ultimately limit the efficacy of these medications [3, 4]. Tolerance is the diminishing response to medication, requiring escalating doses to achieve the same pain relief. Increasing the dose of opioids increases the possibility of adverse side effects including constipation, respiratory depression, and nausea [5, 6]. Conversely, hyperalgesia is hypersensitivity to pain and also can be caused by the administration of opioid medication [6]. The therapeutic limitations of opioid medications in chronic pain management show a clear need to understand the molecular mechanisms which underpin the biologic processes governing chronic pain, tolerance to pharmacologic medicine, and hyperalgesia.

Neuroplasticity in the form of adaptive changes in protein transcription and translation may contribute to the development of chronic pain, opioid tolerance, and its hyperalgesia [7]. Changes in the expression of neuronal nitric oxide synthase (nNOS), protein kinase C (PKC), CaMKIIα, and other proteins are associated with chronic pain conditions and opioid-induced hyperalgesia or tolerance [8–11]. The mechanisms and signaling pathways are still not fully understood, but mammalian target of rapamycin (mTOR) represents a key player in the mechanism governing neuroplasticity in chronic pain and opioid-induced hyperalgesia/tolerance disorders [12–21]. This paper reviews recent evidence regarding the role of mTOR in chronic pain, opioid tolerance, and opioid-induced hyperalgesia and discusses how mTOR participates in the development and maintenance of these disorders. Current evidence suggests that mTOR likely represents an excellent candidate target for novel pharmaceutical intervention in chronic pain, opioid tolerance, and opioid-induced hyperalgesia in patients.

### mTORC1 and mRNA translation

**Fig. 1:**
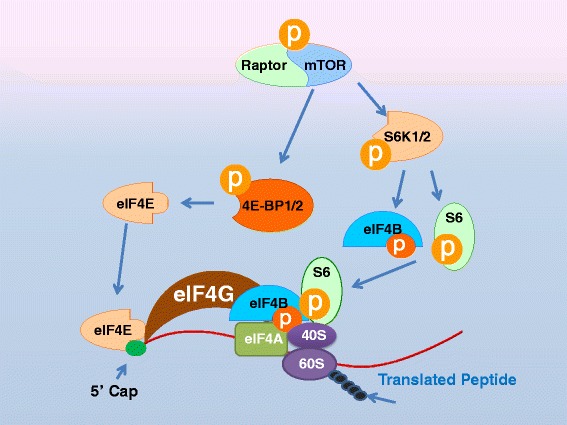
mTOR signaling pathway. Activated mTOR phosphorylates 4E-BP1/2 and S6K1/2. The phosphorylation of 4E-BP1/2 causes the release of eIF4E which is then free to combine with the translation initiation complex, resulting in increased protein translation. Phosphorylated S6K1 phosphorylates eIF4B and S6 which also lead to increased protein translation. 4E-BP1/2: eIF4E-binding protein1/2. 40S: an eukaryotic small ribosomal subunit. 60S: an eukaryotic large ribosomal subunit. eIF4E/B/G/A: eukaryotic translation initiation factor 4E/4B/4G/4A. mTOR: mammalian target of rapamycin. p: phosphorylated. S6K1/2, p70 ribosomal S6 Kinase 1/2

Mammalian target of rapamycin (mTOR) is a serine-threonine protein kinase which forms 2 distinct evolutionary preserved protein complexes known as mTOR complex-1 (mTORC1) and mTOR complex-2 (mTORC2) [22, 23]. Active mTOR bound to Raptor protein forms the rapamycin-sensitive mTORC1 complex that is predominantly responsible for regulating protein translation (Fig. 1) [24]. The mTORC1 regulates the activity of at least 4 proteins involved in protein translation via phosphorylation—4E-BP1/2, eIF4B, S6K1, S6—during the following steps. Most mature eukaryotic mRNAs possess a 7-methyl-guanine cap structure at the 5’-end, which controls initiation of translation [23]. During translation initiation, the cap structure is recognized by the eIF4G initiation complex that includes the eIF4E protein [23]. Under basal conditions, eIF4E remains bound to the eukaryotic initiation factor 4E (eIF4E)-binding protein 1/2 (4E-BP1/2), which prevents formation of eIF4G (Fig. 1) [25]. Active mTOR leads to phosphorylation of 4E-BP1/2, which changes its shape and releases eIF4E, allowing the formation of the functional eIF4G complex and initiation of translation (Fig. 1) [23, 25]. In addition, recruitment of the eukaryotic initiation factor 4B (eIF4B) to the initiation complex is mTOR-dependent [26, 27]. The eIF4B needs to be phosphorylated by the p70 ribosomal S6 protein kinase 1/2 (S6K1/2) to associate with the translation initiation complex (Fig. 1) [26, 27] and mTOR activation also induces phosphorylation of S6K1, thereby activating it. S6K1/2 is better known for kinase activity toward ribosomal S6 proteins (S6) [22, 28, 29], which are critical for stimulating the translation rate of mRNAs containing the 5’-oligopyrimidine tract (Fig. 1). This subgroup of mRNAs encodes primarily the proteins involved in the translation process itself, such as all ribosomal proteins and several elongation factors [30]. Thus, mTOR activation leads to an increase in the translation capacity of the cell.

### mTOR inhibitors in clinical use

Rapamycin, a specific inhibitor of mTOR, was discovered at Easter Island (former Rapa Nui) in a bacterial strain named *Streptomyces hygroscopius* [31]. Rapamycin was first found to inhibit the growth of yeast and was being developed as an antifungal drug [32]. Its anti-fungal use was temporally abandoned after the discovery of its potent immunosuppressive activity, which later proved beneficial for transplant patients [33, 34]. The mTOR inhibitor and some of its derivatives also showed anti-proliferative activity which was found useful in the treatment of certain cancers [31, 35–37]. Studies have shown hyperactivity of mTOR in gliomas [38] as well as nonglial brain tumors [39]. The specificity by which rapamycin and its analogues bind to and inhibit mTORC1 activity thereby abrogating the proliferation of these cancers has prompted clinical trials to investigate the efficacy of rapamycin and its analogues “Rapalogs” as novel treatments in cancer therapy and their approval for specific indications [34, 40, 41]. Rapamycin was recently shown to prolong the life of mice [42], whether this effect occurs in humans is unknown and remains to be determined.

### mTOR expression in pain-related regions

mTOR is expressed and distributed in pain-related central nervous system regions. Xu et al. used immunofluorescence to analyze the distribution of mTOR, 4E-BP1/2, S6K and their phosphorylated counterparts in dorsal root ganglia (DRG) and spinal cord dorsal horn [43]. mTOR was found in approximately 26.1 % of DRG neurons and S6K was found in about 19.1 % of DRG neurons, with most of small diameter [43]. 4E-BP1 was exclusively found in DRG satellite glial cells, but it co-localized in dorsal horn with mTOR and S6K. 4E-BP1, mTOR, and S6K are highly expressed in the superficial dorsal horn [43]. Interestingly, the activated or phosphorylated forms of these proteins were virtually undetectable or at very low levels under normal conditions in the DRG and dorsal horn [43]. These findings support the behavioral observation that intrathecal administration of the mTOR inhibitor rapamycin does not affect basal pain perception, suggesting that mTOR and its downstream effectors do not play a key role in acute pain.

### mTOR in chronic pain

**Fig. 2:**
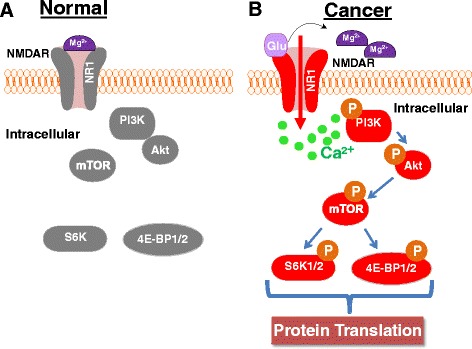
Proposed mechanism of spinal cord NMDA receptor-mediated activation of mTOR signaling in cancer pain. Under normal conditions (**a**), magnesium blocks NMDAR activity, thus silencing the intracellular kinases including the mTOR signaling pathway. Under cancer conditions (**b**), cancer-caused noxious insult leads to removal of the magnesium from NMDA receptors, resulting in calcium influx through NMDA receptor activation. The influx of calcium may then activate PI3K and Akt kinases which go on to phosphorylate mTOR. Active mTOR phosphorylates S6K1/2 and 4E-BP1/2 leading to protein translation initiation. 4E-BP1/2: eIF4E-binding protein1/2. Akt: protein kinase B. mTOR: mammalian target of rapamycin. NMDAR: NMDA receptor; NR1: a subunit of NMDA receptors. p: phosphorylated. PI3K: phosphoinositide 3-kinase. S6K1/2, p70 ribosomal S6 Kinase 1/2

mTOR’s role in cancer treatment is not simply limited to its effects on neoplastic cell survival and proliferation. Studies have shown that activation of mTOR and its downstream effectors in spinal cord (but not in DRG) are implicated in cancer pain [18, 44]. Shih et al. showed that rats injected with prostate cancer cells into the tibia, a model of bone cancer pain, experienced pain hypersensitivity [18]. This hypersensitivity was attenuated following intrathecal injection of rapamycin [18]. Rapamycin’s effect is dose-dependent without affecting locomotor function and without significant systemic side effects such as immunosuppression [18]. Furthermore, they showed that levels of phosphorylated mTOR (p-mTOR) and p-S6K increased in the L4-5 dorsal horn and DRG on the side of the prostate cancer cell injection [18]. This increase in p-mTOR and p-S6K was blocked in the presence of an NMDA receptor antagonist [18]. The authors proposed that the activation of NMDA receptor-mediated spinal cord mTOR pathways contribute to the initiation, establishment, and maintenance of bone cancer-induced pain hypersensitivity [18] (Fig. 2). This conclusion is further supported by the observation that NMDA receptor subunit NR1 co-localized with mTOR and S6K in dorsal horn neurons [18].

Spinal cord mTOR and its downstream pathway also are involved in inflammatory pain. Liang et al. showed that intraplantar injection of complete Freund’s adjuvant (CFA) in a model of chronic inflammatory pain increased the levels of p-mTOR and p-S6K1 in the ipsilateral L4/5 spinal cord and DRG [16]. Behavioral testing demonstrated that CFA-induced mechanical and thermal pain hypersensitivity could be alleviated by intrathecal administered rapamycin [16]. Additionally, Xu et al., used λ-carrageenan to induce persistent peripheral inflammation in rats and found increased levels of not only p-mTOR but also upstream phosphorylated protein kinase B (Akt) in dorsal horn [20]. The downstream targets of mTOR activation, p-S6K and p-4E-BP1 were also elevated in dorsal horn [20]. More importantly, intrathecal administration of rapamycin produced anti-nociceptive effects in this persistent inflammatory pain model [20]. These anti-nociceptive effects of rapamycin were also observed in the second phase of the formalin model [20]. Selective inhibitors of PI3K (upstream of Akt), Akt, or mTORC1 attenuated phase II flinching behavior in rats that were injected with formalin [20]. Phase II of formalin-induced inflammation is known to represent spinal sensitization [45]. These findings demonstrate the potential highly targeted role of mTOR inhibitors in the treatment of inflammatory pain.

Although the mechanism of mTOR-induced inflammatory pain sensitivity is relatively clear, how mTOR is involved in neuropathic pain remains elusive. Rapamycin administration in neuropathic pain models attenuates pain hypersensitivity in several studies in both rats and mice [17, 18, 46]. Géranton et al. reported that spared nerve injury (SNI)-induced mechanical allodynia was attenuated by rapamycin delivered intrathecally 6 days after surgery [47]. However, western blotting analysis of dorsal horn and dorsal roots 7 days after SNI revealed no significant changes in the expression of p-S6K [47]. Additionally, immunostaining showed no change in the percentage of peripherin-labeled fibers expressing p-mTOR [47]. Liang et al. also showed no change in the basal level of p-mTOR in spinal cord and DRG after spinal nerve ligation [16]. Conversely, Zhang et al. used the chronic constriction injury (CCI) model of neuropathic pain to analyze the role of mTOR in neuropathic pain [21]. The phosphorylated counterparts of mTOR, 4E-BP1, and S6K were upregulated in the spinal cord 7 days and 14 days after CCI [21]. Intrathecal rapamycin not only blocked this upregulation but also attenuated CCI-induced mechanical allodynia (but not thermal hyperalgesia) [21]. These data suggest that distinct types of peripheral nerve injury differentially may regulate the activation of mTOR and its downstream effectors in spinal cord and DRG. Further research into the mechanism of rapamycin antinociception in neuropathic pain is required.

### mTOR in opioid-induced tolerance and hyperalgesia

Evidence has shown that opiate-induced tolerance and hyperalgesia may be attributed to changes in the transcription and translation of several key tolerance-associated proteins including neuronal NOS, PKCγ, and CaMKIIα in the central nervous system as well as in the peripheral nervous system [3, 48–51]. Given that mTOR regulates protein translation, it is reasonable to assume that mTOR participates in the development and maintenance of opioid-induced tolerance and hyperalgesia. Indeed, Xu et al**.** used an animal model of opioid tolerance/hyperalgesia: twice-daily intrathecal injections of 10 μg of morphine for 6 continuous days produced a time-dependent decrease in morphine’s maximal potential analgesic effect (MPAE) at 3, 5, and 7 days post morphine injection [19]. Reductions in mechanical threshold and thermal latency occurred at 8 days post morphine injection [19]. Rapamycin administered intrathecally before or after morphine treatment blocked a decrease in morphine's MPAE, and attenuated morphine’s effect on mechanical threshold and thermal latency [19]. Similar to the effect of rapamycin, intrathecal administration of siRNA specific for mTOR before morphine attenuated the tolerance and hyperalgesia [19].

Xu et al. further demonstrated that mTOR and its downstream effectors is activated by repeated morphine injections through the μ opioid receptor-triggered P13K/Akt pathway in dorsal horn neurons of the spinal cord [19]. A PI3K or Akt specific inhibitor prevented morphine induced increases in the phosphorylated forms of 4E-BP1, mTOR, and S6K1 in the spinal cord. These inhibitors also attenuated behavioral responses observed in the opioid-induced tolerance and hyperalgesia model [19]. In contrast, basal pain perception and locomotor functioning were left untouched. These findings indicate that PI3K and Akt are involved in mTOR’s effect on opioid-induced tolerance and hyperalgesia but not basal pain perception and locomotor functioning.

**Fig. 3:**
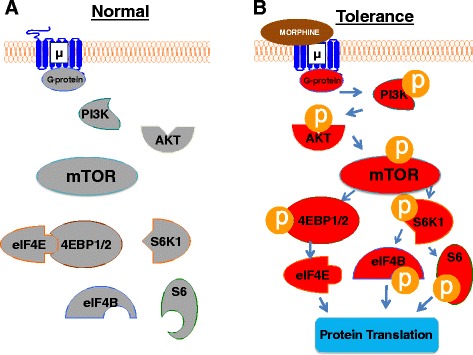
Proposed model for the involvement of spinal cord mTOR in chronic opioid tolerance and hyperalgesia. Under normal conditions (**a**), mTOR is inactivated. 4E-BP1/2 binds to eIF4E, preventing its associations with the translation initiation complex, thus silencing protein translation. After repeated morphine exposure (**b**), activation of μ opioid receptors leads to the phosphorylation of PI3K and Akt, initiating a phosphorylation cascade including mTOR/S6K1/2/4E-BP1/2. eIF4E release, phosphorylated eIF4B, and phosphorylated S6 trigger protein translation. 4E-BP1/2: eIF4E-binding protein1/2. Akt: protein kinase B. eIF4E/eIF4B, eukaryotic translation initiation factor 4E/4B. mTOR: mammalian target of rapamycin. p: phosphorylated. PI3K: phosphoinositide 3-kinase. S6K1/2, p70 ribosomal S6 Kinase 1/2. S6K1, S6 Kinase 1. μ: μ opioid receptor

To further uncover the mTOR-dependent mechanism (s) of opioid-induced tolerance and hyperalgesia, Xu et al. observed protein translation in their model of morphine-induced tolerance [19]. Repeated morphine injections increased nascent protein synthesis as determined by L-azidohomoalanie. Rapamycin co-administered with morphine diminished morphine’s increase in nascent protein synthesis. The binding of eIF4A to eIF4E, components of the translation initiation complex, increased in dorsal horn after repeated morphine exposure, but this increase was attenuated following rapamycin co-injection. Rapamycin also attenuated the increases of several tolerance-associated proteins including PKCγ, nNOS, and CaMKIIα. These findings provide a mechanism of opioid-induced tolerance and hyperalgesia in which μ opioid receptor activation activates PI3K/Akt which triggers an mTOR-dependent signaling cascade that results in increased protein translation (Fig. 3) [19].

## Conclusion

The significance of mTOR’s function in the grand scheme of pain medicine is undisputed. mTOR is a key puzzle piece which contributes to the understanding of many aspects of the science of chronic pain. Numerous potential therapeutic options exist in rapamycin and its analogues, given mTOR’s versatility in function in many aspects of pain mediation, cancer, and post-transplant immunosuppression. mTOR is involved in cancer pathogenesis, and mTOR inhibitors are currently being used as antineoplastic agents. New data demonstrates that mTOR shows promise as a target for chronic inflammatory pain, neuropathic pain, as well as cancer pain. Recent data also delivers cutting edge science tying the vital role mTOR has in the acquisition and maintenance of opioid tolerance/hyperalgesia. These findings prompt us to investigate the applicability of mTOR-targeting therapies for future treatment of a vast cadre of chronic pain syndromes.

### Summary statement

This review highlights recent findings regarding the role of mTOR and its downstream signals in pain-related regions after persistent inflammation, nerve injury, or repeated opioid injections and it discusses how mTOR participates in the development and maintenance of chronic pain and opioid-induced tolerance and hyperalgesia.

## Abbreviations

4E-BP1/2: Eukaryotic initiation factor 4E (eIF4E)-binding protein 1/2
DRG: /Akt, protein kinase B dorsal root ganglia
eIF4A: Eukaryotic initiation factor 4A
eIF4B: Eukaryotic initiation factor 4B
eIF4G: Eukaryotic initiation factor 4G
mTOR: Mammalian target of rapamycin
mTORC1: MTOR complex-1
mTORC2: MTOR complex-2
NMDA: N-Methyl-D-aspartate
NMDAR: Receptor for N-Methyl-D-aspartate
NNOS: Neuronal nitric oxide synthase
NR1: A subunit of NMDA receptors
PI3K: Phosphoinositide 3-kinase
PKC: Protein kinase C
S6K1: P70 ribosomal S6 protein kinase 1/2
S6: P70 ribosomal protein S6
